# Evaluation of using micronized saudi calcite in ilmenite-weighted water-based drilling fluid

**DOI:** 10.1038/s41598-024-63839-6

**Published:** 2024-06-04

**Authors:** Amir Shokry, Salem Basfar, Salaheldin Elkatatny

**Affiliations:** Department of Petroleum Engineering, College of Petroleum Engineering & Geosciences, King Fahd University of Petroleum & Minerals, 31261 Dhahran, Saudi Arabia

**Keywords:** Micronized saudi calcite, Sagging, Rheological parameters, Water-based drilling fluid, Filtration performance, Ilmenite, Chemistry, Engineering

## Abstract

A high-density water-based drilling fluid (WBDF) is crucial for maintaining wellbore stability, controlling formation pressures, and optimizing drilling performance in challenging subsurface conditions. In the present research, the effect of micronized calcium carbonate (calcite), extracted from the Aruma formation outcrop, is evaluated as one of the additives that could be added to the ilmenite-weighted WBDF to enhance and optimize its properties. Various concentrations of Calcite microparticles were introduced into identical fluid formulations to assess their impact. The concentrations ranged from 0, 10, 20, to 30 lb/bbl, providing a comprehensive examination of the effects of calcite microparticles across a spectrum of concentrations within the fluid. The results highlighted that adding Barite microparticles to the WBDF revealed a notable enhancement in rheological properties. Specifically, the yield point demonstrated an increase of 37%, 37%, and 11% for concentrations of 10, 20, and 30 lb/bbl of calcite, respectively. Equally significant, high-pressure-high-temperature (HPHT) filtration analysis indicated a considerable enhancement for the fluids containing calcite microparticles. A reduction of 14.5%, 24.6%, and 13% were observed in HPHT filtrate for concentrations of 10 lb/bbl, 20 lb/bbl, and 30 lb/bbl respectively. Simultaneously, there is a reduction in filter cake thickness by 20%, 40%, and 20%, respectively. No ilmenite settling was observed in the sample containing 20 lb/bbl of calcite, unlike the other concentrations. These diverse results strongly suggest that the optimal concentration for calcite microparticles is 20 lb/bbl. The combined utilization of the optimal concentration of calcite microparticles alongside the established additives proves to be an effective strategy for optimizing the ilmenite-weighted WBDF performance in terms of both thermal stability and rheological behavior.

## Introduction

Drilling fluids are indispensable in oil well drilling, playing a crucial role in maintaining wellbore stability, controlling pressures, and carrying cuttings to the surface^1–3^. Beyond facilitating the drilling process, these fluids serve as coolants, preventing equipment overheating, and create a protective barrier to prevent wellbore wall collapse. Their versatility extends to improving drilling rates, ensuring safety, and contributing to the economic viability of oil extraction. In essence, drilling fluids are essential for successfully navigating the technical challenges and environmental considerations inherent in oil well drilling^4,5^.

Water-based drilling fluids (WBDF) and oil-based drilling fluids (OBDF) represent two distinct categories in the realm of drilling operations, each tailored to specific geological and operational requirements. Water-based drilling fluids, being environmentally friendly, predominantly consist of water, various clays, and additives. These fluids excel in formations where water can be effectively utilized, providing cost-efficiency and ease of disposal. OBDF, composed of oil, emulsifiers, and other additives, finds prominence in challenging environments such as high-temperature formations and when encountering hydrophobic formations. These fluids offer superior lubrication, thermal stability, and reduced formation damage, making them ideal for specific drilling conditions. The choice between WBDF and OBDF hinges on a careful consideration of geological characteristics, environmental impact, and operational demands, underscoring the importance of selecting the most suitable fluid for optimal drilling performance^6,7^.

The drilling fluid plays a vital role in safeguarding reservoir integrity during oil and gas exploration and development by minimizing invasion, preserving wellbore stability, and ensuring overall reservoir integrity^8,9^. Its primary function involves creating a protective barrier to mitigate formation damage, facilitating efficient resource discovery, appraisal, and development^10,11^. One of the main factors that significantly influences formation damage is the particle size of solid material in the drilling fluids, with improper sizing potentially leading to pore space clogging within the reservoir rock. Maintaining an optimal particle size distribution is crucial to ensure compatibility with the pore size distribution, preventing impediments to fluid flow and preserving the reservoir's permeability throughout drilling operations^12–15^.

The incorporation of nanoparticles in WBDF represents an innovative approach that holds significant promise in enhancing various aspects of drilling operations^9,16^. Nanoparticles, owing to their minute size and high surface area, can impart unique properties to the drilling fluid. These properties include improved rheological behavior^17,18^, enhanced thermal stability^19^, and increased lubricity^20,21^. Nanoparticles can play a role in minimizing fluid invasion into the formation, reducing the potential for formation damage^22,23^. Their presence can also contribute to the overall stability of the wellbore, preventing issues such as wellbore collapse and improving the efficiency of cuttings transport to the surface^24^. The utilization of nanoparticles in WBDF signifies a frontier in drilling technology, demonstrating potential advancements in fluid performance and overall drilling efficiency^25^.

The particle size in drilling fluids plays a critical role in potential formation damage, as improper sizing, especially when it aligns with the pore throat dimensions, can lead to invasion and reduced permeability, underlining the importance of utilizing the correct particle size distribution to safeguard porous formations during drilling operations^26,27^. When choosing an appropriate particle size for use as a bridging material, it is crucial to avoid selecting particles within the same range as the formation permeability. If the particles align with the pore throat diameter, as demonstrated in experiments using a ceramic disk with a mean pore throat diameter of 53 µm and fluid particles of approximately 50 µm, it can lead to a considered decrease in permeability, confirming a reported decrease of 17–18%^28^. numerous researchers affirm that the particle size should ideally be about one-third of the rock pore throat^12^.

The application of calcite nanoparticles in drilling fluids represents a contemporary and promising advancement in drilling technology^29^. These nanoparticles, owing to their compact dimensions, abundance, cost-effectiveness, and safety, have gained attention as attractive additives in WBDF^30^. Recent studies have explored their incorporation into bentonite water-based drilling fluids, ranging from 0.025 to 0.5% concentrations. The introduction of calcite nanoparticles has exhibited noteworthy effects on filtering characteristics and the generation of thinner cake surfaces^31^. Researchers have investigated their impact on mitigating drilling fluid absorption into shale matrices, achieving substantial permeability decreases of up to 95.5% at a nanoparticle concentration of 1%^32^. Although the encouraging results, different factors remain to be addressed concerning the rheological and viscoelastic properties of those fluids, as well as concerns regarding the settling of the weighting materials to prevent issues such as compromised fluid stability and diminished drilling performance. Further research is essential to gain a deeper understanding and refine the optimization of calcite nanoparticles and microparticle concentration within water-based mud to reach the most stable drilling fluids properties.

This study is dedicated to examining the impact of incorporating Calcite microparticles into ilmenite-weighted WBDF at varying concentrations ranging from 0, 10, 20, to 30 lb/bbl. The ultimate objective is to determine the optimum concentration that ensures a stable fluid, eliminating ilmenite sagging issues, and simultaneously achieving controlled filtration properties and improved rheological characteristics. The study explores the process of preparing micronized calcite sourced from the Aruma Saudi formation, providing a comprehensive evaluation of this material through a range of characterization techniques, including particle size distribution (PSD), X-ray diffraction (XRD), and scanning electron microscopy (SEM).

## Methodology and materials

### Calcite microparticles preparation

**Figure 1:**
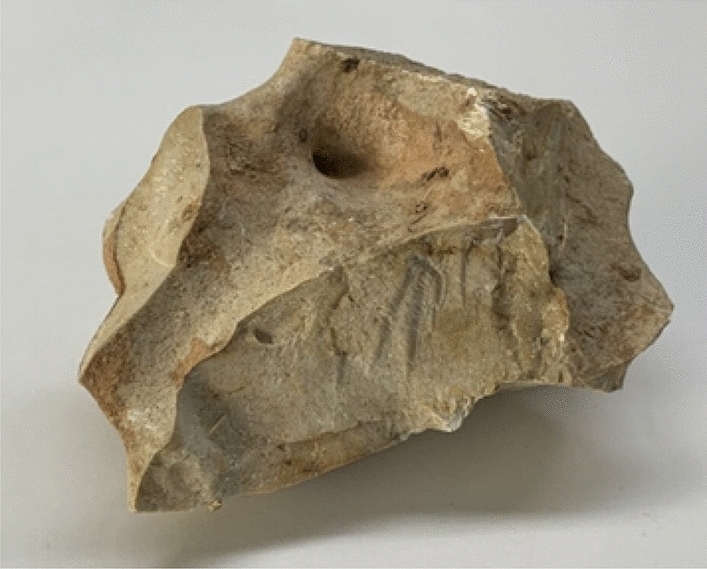
Limestone of optical quality extracted from the Aruma formation.

The preparation of calcite microparticles encompasses a methodical procedure designed to generate precisely tuned particles tailored for specific applications. Initially, the calcite particles were extracted from the Khanasir limestone member within the Aruma Saudi formation. The Aruma outcrop, situated in the central region near Al-Kharj City in Saudi Arabia, exhibits a distinctive lithology characterized by its prominent limestone formations, including the notable Khanasir member^33,34^. Employing a ball milling machine, the limestone rock illustrated in Fig. 1 underwent crushing and subsequent sieving to ensure a homogeneous microparticle size, with a particle diameter not exceeding 10 µm^30^.

### Analysis of calcite microparticle characteristics

The analysis of calcite microparticle characteristics involves a comprehensive examination aimed at understanding and documenting various key attributes of these particles. Through techniques such as microscopy, spectroscopy, and particle size analysis, researchers can delve into the morphology, composition, and size distribution of calcite microparticles. Precise measurements of particle size distribution contribute to a thorough understanding of the uniformity and size variations within the calcite microparticle sample.

#### Particle size distribution

The HELOS particle size analyzer was employed to examine the distribution of the particle size for the three distinct: ilmenite, conventional calcite (50 µm), and sieved calcite. This analytical approach provides a detailed and quantitative assessment of the size distribution of particles within the respective samples.

**Figure 2:**
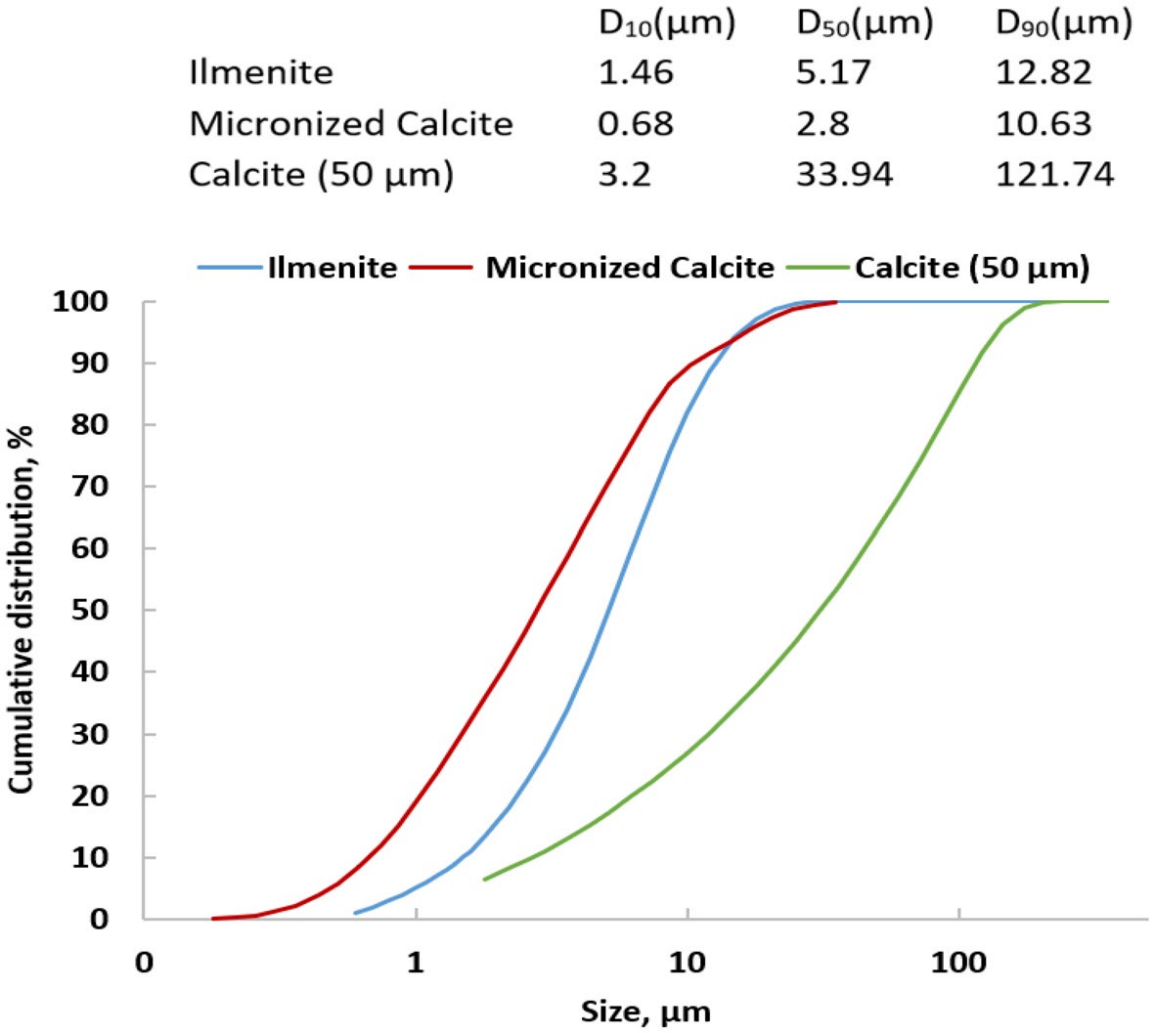
Particle size distribution for ilmenite, micronized calcite, and conventional 50 µm calcite.

The test results for the three samples indicate that ilmenite aligns closely with micronized calcite in terms of a tight distribution range, in contrast to normal calcite with a particle size of 50 microns, displaying a broader distribution range as depicted in Fig. 2. Ilmenite and micronized calcite exhibit D_50_ values of 5.17 µm and 2.8 µm, respectively, while normal calcite has a D_50_ value of 33.94 µm. This implies that a combination of ilmenite and micronized calcite would be optimal, given the minimal variation in size distribution for both. Notably, The D_50_ value of micronized calcite particles is smaller than that of ilmenite, which suggests that incorporating micronized calcite can enhance the rheological properties and other parameters of the ilmenite-weighted WBDF, offering a promising prospect for improved performance in various applications.

#### Scanning electron microscopy (SEM)

**Figure 3:**
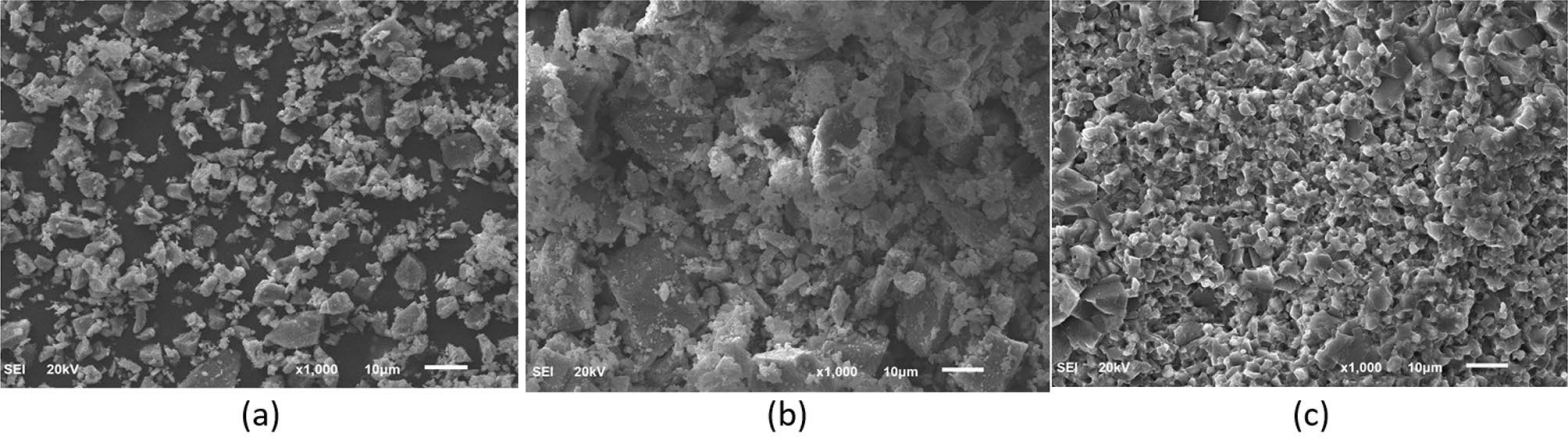
SEM of Ilmenite (**a**), calcite (50 µm) (**b**), and micronized calcite (**c**).

Scanning electron microscopy was utilized to scrutinize the morphology of the three samples, and the outcomes are visually represented in Fig. 3. Figure 3a represents the SEM of ilmenite which is the weighting material of this drilling fluid used in this study. Figure 3b represents the SEM of the conventional form of 50 µm calcite in which a lack of uniformity is apparent in both particle size and shape. The predominant morphology in this sample leans towards an angular to sub-angular structure. However, Fig. 3c represents a micronized form of calcite that exhibits a more consistent size and, to a certain extent, shape. A noteworthy characteristic is the prevalence of sub-rounded to rounded shapes among the majority of micronized calcite particles.

In conclusion, the scanning electron microscopy results highlight significant differences in particle morphology among the conventional and micronized forms of calcite. Conventional calcite exhibits irregular sizes and angular shapes, micronized calcite stands out with a more uniform particle size distribution and a tendency towards sub-rounded to rounded shapes. These distinctions in particle characteristics are crucial considerations in understanding the potential applications and performance of these materials in various industries, offering insights for further optimization and utilization in specific contexts.

#### The X-ray diffraction (XRD) and X-ray fluorescence (XRF)

**Figure 4:**
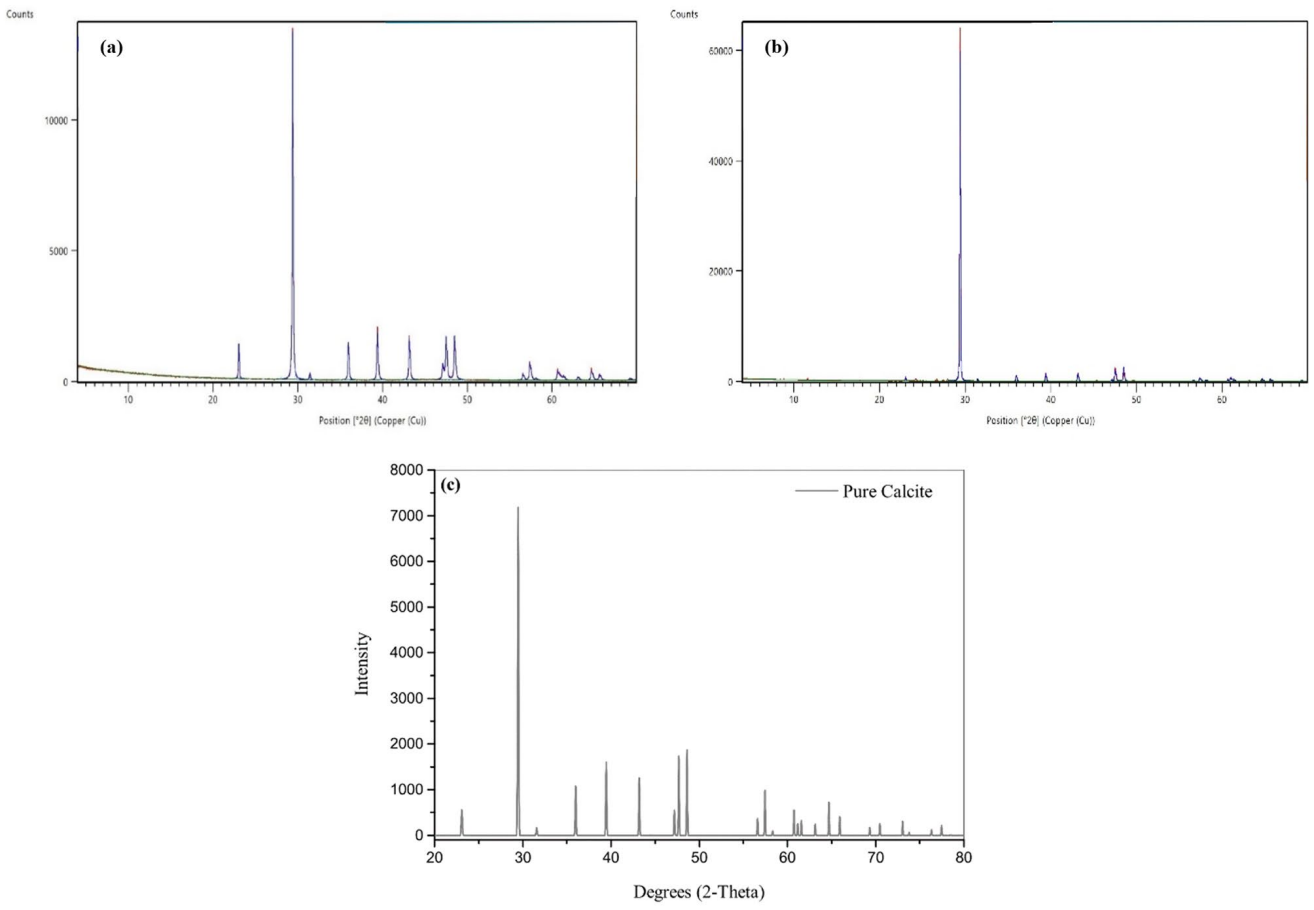
X-ray diffraction pattern of micronized calcite (**a**), commercial 50 µm calcite (**b**), and pure calcite from literature^35^.

The analysis of crushed limestone rock using X-ray diffraction (XRD) is a crucial technique for confirming its composition, which is mainly calcium carbonate (calcite). After carefully preparing the samples, which involves crushing the limestone into a fine powder to ensure uniformity, the XRD experimental setup utilizes a powerful X-ray source and a detector positioned to capture the diffracted rays. By methodically scanning the 2θ angles, the XRD pattern obtained displays discernible peaks that correspond to the crystallographic structure of the minerals found. After careful examination, it has been determined that the sample shown in Fig. 4a contains only calcite mineral based on the identification of characteristic peaks of 23.03, 29.37, 35.96, 39.39, 43.05, 47.5, and 57.45° which closely resemble those present in commercial 50 µm calcite and pure calcite reference patterns showcased in Fig. 4b,c^35^. Micronized calcite and conventional 50µm calcite composition analyses using X-ray Fluorescence (XRF) are displayed in Table 1. The table clearly illustrates that micronized calcite is primarily composed of calcium (Ca), accounting for 97 wt%, with minor amounts of other traces. conventional 50µm calcite also consists primarily of calcium (85 wt%), aluminum (7.55 wt%), and silicon (6.65 wt%), with small amounts of iron and sulfur present.

**Table 1:** XRF for calcite (50 µm), and calcite microparticles.

Component	Cacite (50 µm), wt%	Micronized calcite, wt%
Ca	85	97
Si	6.65	0.41
Fe	1.55	1.43
Al	7.55	0.65
S	0.22	0.17

### Drilling fluid formulations

An ilmenite-weighted WBDF was meticulously prepared following the mud formulation outlined in Table 2. The formulation served as a foundational fluid to facilitate the examination of the effects of micronized calcite when introduced at various concentrations. The mixing sequence, as specified in Table 2, was rigorously adhered to across all samples to ensure consistent and indicative results. The formulation process commenced with the addition of soda ash to the mixing water, aiming to regulate the hardness within the accepted range for subsequent additive reactions. A minor quantity of defoamer was then incorporated into the recipe to mitigate the potential generation of foams. Subsequently, the fluid viscosity was adjusted with Barazan D-plus, a high-molecular-weight polysaccharide biopolymer. Fluid loss control was achieved through the addition of PAC-LV, a polyanionic cellulose polymer, and Dextrid, a modified and bacterially stabilized starch. Sodium hydroxide was introduced to regulate pH within the accepted range, followed by the addition of potassium chloride to enhance clay stabilization. The desired mud density was attained by incorporating the necessary quantity of ilmenite. These additives underwent thorough mixing at ambient room conditions using a three-speed mixer.

**Table 2:** The formulation for the drilling fluid composition, (lb/bbl) = (g/350 cm^3^).

Component	Quantity	Unit	Mixing time, min	Function
Water	0.552	bbl	–	Base fluid
Soda ash	0.5	lb/bbl	2	Hardness controler
Defoamer	0.1	gal/bbl	2	Defoamer agent
Barazan D Plus	0.75	lb/bbl	20	Rheology modifier
PAC-LV	2	lb/bbl	10	Filtration control and shale encapsulator
Dexteride	5	lb/bbl	10	HT fluid loss reducer
NaOH	0.6	lb/bbl	2	Alkalinity control
KCl	20	lb/bbl	5	Potassium source and shale inhibitor
Ilmenite	250	lb/bbl	15	Weighting material
Micronized calcite	0, 10, 20, 30	lb/bbl	10	Micronized particles (new material)

### Drilling fluid lab tests

For an initial assessment of the base fluid, a series of drilling fluid lab tests was executed. The evaluation encompassed pH measurement, determination of mud weight, followed by subjecting the sample to 250° F hot rolling conditions for 16 h to simulate well conditions and assess sagging properties. Subsequent evaluations after the hot rolling process encompassed rheological properties and HPHT (high-pressure high-temperature) filtration parameters.

Following the confirmation of the stability of the base fluid, the introduction of micronized calcite particles ensued, incorporating various concentrations into the base fluid. This deliberate addition aimed to systematically assess and analyze the impact of these particles on diverse properties associated with the fluid. The investigation encompasses an exploration of how varying concentrations of micronized calcite influence different characteristics and performance parameters of the fluid, providing valuable insights into the interaction dynamics and potential enhancements or modifications resulting from the addition of these particles.

#### The rheological properties

The Fann M3600 viscometer is employed to measure rheological properties using the Bingham model through a straightforward process. Fluid samples are subjected to controlled shear rates, allowing for the determination of key rheological parameters. The Bingham model, specifically designed for materials with yield stress like drilling fluids, aids in unveiling essential information such as yield point and viscosity.

After subjecting the fluid to various shear stresses and recording corresponding readings, the Bingham model, characterized by Eqs. (1)–(3), is utilized. These equations enable the determination of key rheological parameters like plastic viscosity (PV), apparent viscosity (AV), and yield point (YP)^36,37^. Notably, the viscosity is measured in centipoise, while gel strength and yield point are expressed in lb/100ft^2^. This systematic approach provides a quantitative analysis of the fluid’s response to applied stress, shedding light on its resistance to flow, overall viscosity, and the stress required for initiation of movement.

1 $${\text{PV }} = {\text{ RPM}}_{{{6}00}} - {\text{ RPM}}_{{{3}00}} ,$$

2 $${\text{YP }} = {\text{ RPM}}_{{{3}00}} - {\text{PV}},$$

3 $$\text{AV }=\frac{{\text{RPM}}_{600}}{2}.$$

#### Filtration properties

HPHT filtration tests are conducted through the HPHT rheometer, a specialized apparatus designed to simulate downhole conditions in oil and gas wells. This equipment is crucial for evaluating the performance and stability of drilling fluids and other materials under extreme environmental conditions. The HPHT rheometer enables the replication of elevated pressures and temperatures encountered in deep wellbores, allowing to assessment of the thermal and rheological properties of fluids in addition to additives.

The test conditions involve subjecting the system to 250° F, while maintaining a differential pressure of 300 psi across a standard filter paper acting as a filtration medium, having a mean pore diameter of 2–5 µm. Following the API duration of 30 min, during which the filtrate is collected, the obtained volume is multiplied by two^38^. This adjustment accounts for the fact that the used cell has half the standard API cell volume, ensuring a consistent base for comparison purposes. Subsequently, the filtration properties are thoroughly assessed, and the formed filter cake is measured, with its stiffness carefully evaluated. This comprehensive testing approach provides insights into how the drilling fluid behaves under specific temperature and pressure conditions, contributing valuable information for optimizing fluid formulations in high-pressure, high-temperature environments.

#### Static sagging

Understanding static sag is crucial in the realm of drilling fluid analysis as it provides insights into the fluid’s stability and its propensity to maintain its structural integrity over time. This characteristic is particularly important in scenarios where drilling operations may involve intermittent periods of inactivity. Evaluating static sag aids in predicting how well a drilling fluid will perform during these idle periods and contributes to the formulation of fluids that offer consistent and reliable behavior under varying operational conditions in the field^39^.

The sag factor is a quantitative measure of a drilling fluid’s stability, specifically its resistance to deformation during periods of rest or low shear. A lower sag factor indicates better structural integrity, highlighting the fluid’s ability to maintain viscosity and prevent excessive settling or separation over time^40^. The sag factor was determined by applying Eq. (4) under test conditions of 300 psi across the cell at a temperature of 250° F for a duration of 24 h.

4 $$SF=\frac{{\rho }_{bottom}}{{\rho }_{bottom}+ {\rho }_{top}}.$$

The ${\rho }_{bottom}$ represents the density of the fluid at the lower part, while ${\rho }_{top}$ corresponds to the density of the fluid at the top part of the aging cell. Various researchers propose a cut-off value of 0.53 to determine the likelihood of sagging. Values obtained below this threshold are preferred, indicating a lower probability of weighting materials sagging within the fluid^41^.

## Results and discussion

### Density and pH

**Figure 5:**
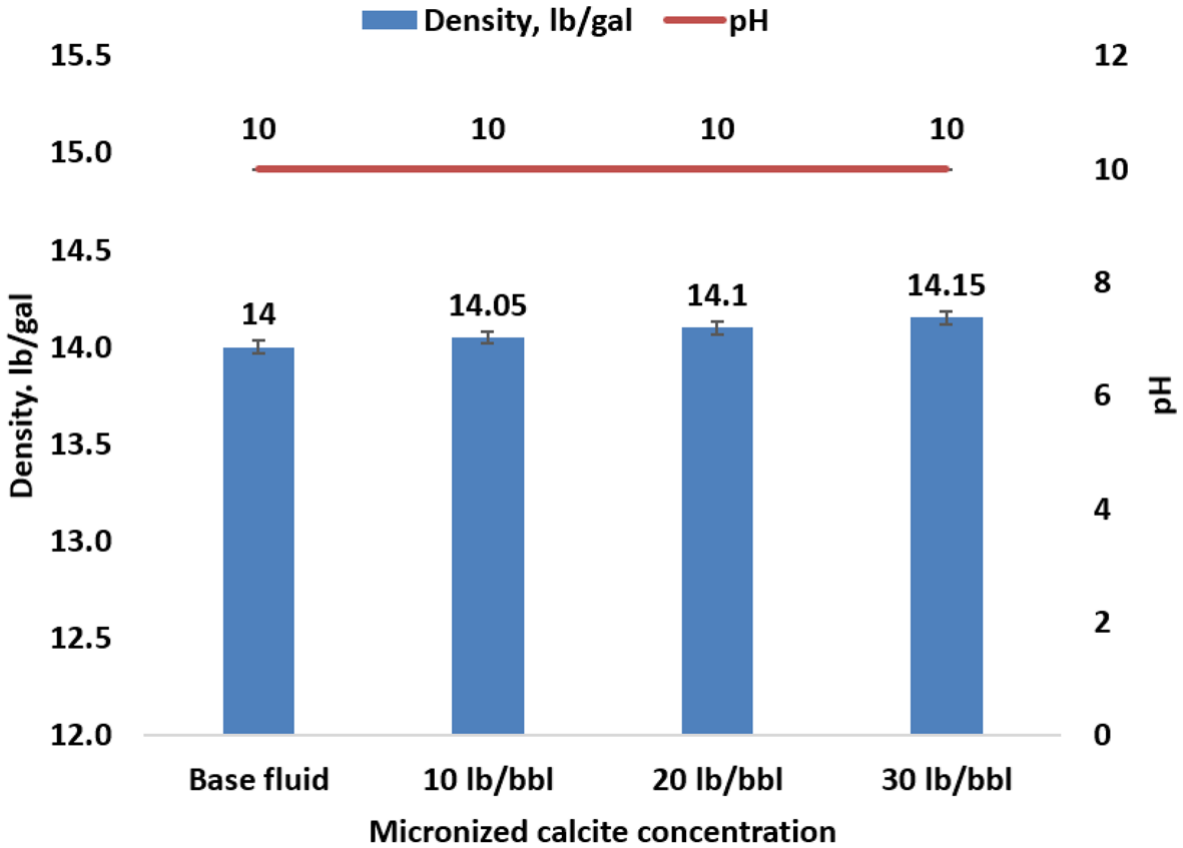
Microparticle calcite effect on the fluid density and pH.

The base fluid, prepared according to the recipe outlined in Table 2, exhibits a mud weight of 14 pounds per gallon (ppg). This elevated mud weight is intentional, tailored to render the fluid suitable for HPHT well conditions. The introduction of varying concentrations of micronized calcite has a negligible impact on fluid density, as depicted in Fig. 5. This minimal effect is attributed to the lower specific gravity of micronized calcite (2.75) compared to ilmenite (4.70–4.79), coupled with the relatively modest quantities of micronized particles added. The total mud weight increases after incorporating 30 lb/bbl of micronized particles is merely 1.1%. An alkalinity test conducted on the base fluid and three other samples with varying concentrations of micronized particles reveals consistent pH values across all fluids as shown in Fig. 5. This indicates that adding micronized particles does not further affect alkalinity. The reason behind this lies in the fact that calcite lacks the high buffering capacity of NaOH, and since it does not readily dissolve in the fluid, it remains inert, contributing to a neutral pH.

### The rheological properties

**Figure 6:**
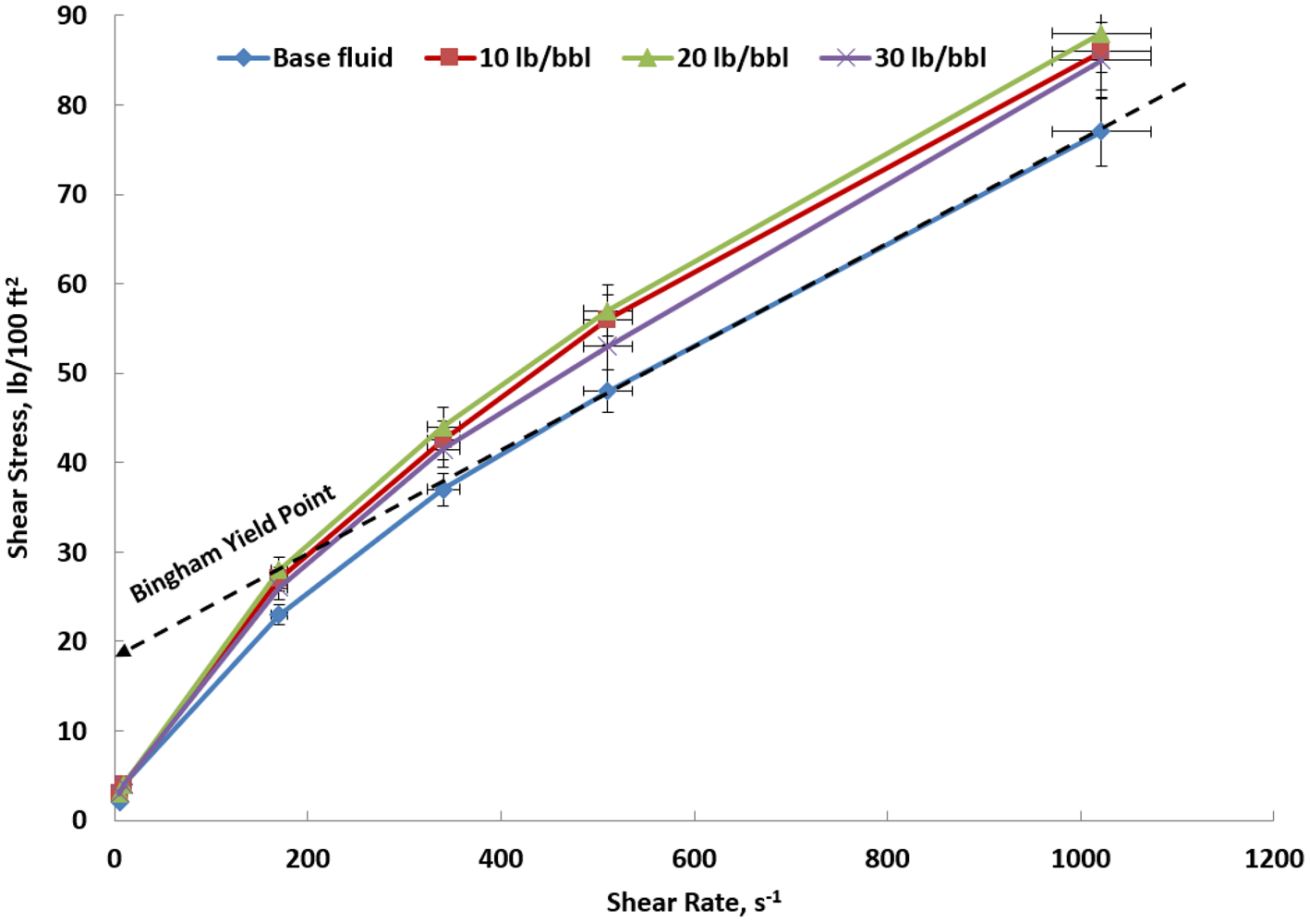
Shear stress reading obtained from the Fann M3600 viscometer is graphed against the corresponding shear rate after hot rolling for 16 h for different calcite microparticle concentrations.

Each shear stress reading obtained from the Fann M3600 viscometer is graphed against the corresponding shear rate for both the base fluid and samples containing different micronized calcite particles, as illustrated in Fig. 6. Generally, increasing the concentration of micronized calcite particles results in a rise in shear stress readings in contrast to the reference fluid. The relationship between the concentration of micronized particles and the increased shear stress is proportional until a critical saturation point is reached. Beyond this point, further increases in concentration lead to a decrease in shear stress. This phenomenon can be attributed to the escalating friction between micronized calcite particles, causing shear stress values to rise until critical saturation is reached. Subsequently, the particles settle at the bottom of the cylinder during shearing, rendering the observed shear stress values misleading. To address this, it is crucial to acknowledge the sagging phenomenon and align with those values in the analysis.

**Figure 7:**
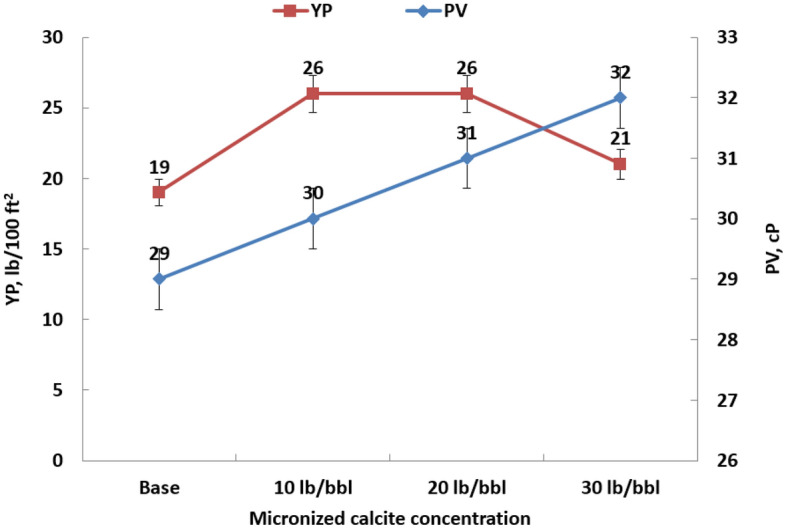
Fluid rheological properties after hot rolling for 16 h for different calcite microparticle concentrations.

The yield point can be determined by employing a Bingham plastic model, which involves considering a straight line at high shear stress, as illustrated in the base fluid in Fig. 6. The intersection of the straight line with the y-axis indicates a yield point of 19 lb/100 ft^2^ for the base fluid. Figure 7 illustrates the Bingham yield points and plastic viscosity for each sample. The observable trend indicates a clear elevation in plastic viscosity with the gradual addition of micronized calcite, given the non-soluble nature of these particles.

It's essential to highlight that the yield point profile diverges from the trend observed in plastic viscosity. While there is a noticeable enhancement in the yield point within the concentration range of 10 lb/bbl to 20 lb/bbl, beyond this point, the yield point starts to decrease. The determining factors for the optimum concentration are influenced by other parameters such as filtration and sagging issues, which will be explored further in the subsequent discussion.

Incorporating the evaluation of gel strength, it is notable that the values remain consistent across different concentrations, except at 20 lb/bbl of micronized calcite. At this concentration, a 25% enhancement is observed in the 10-s reading. This enhancement aligns with a flat rheology, signifying that there is no gradual increase in gel strength beyond this point.

### The filtration properties

**Figure 8:**
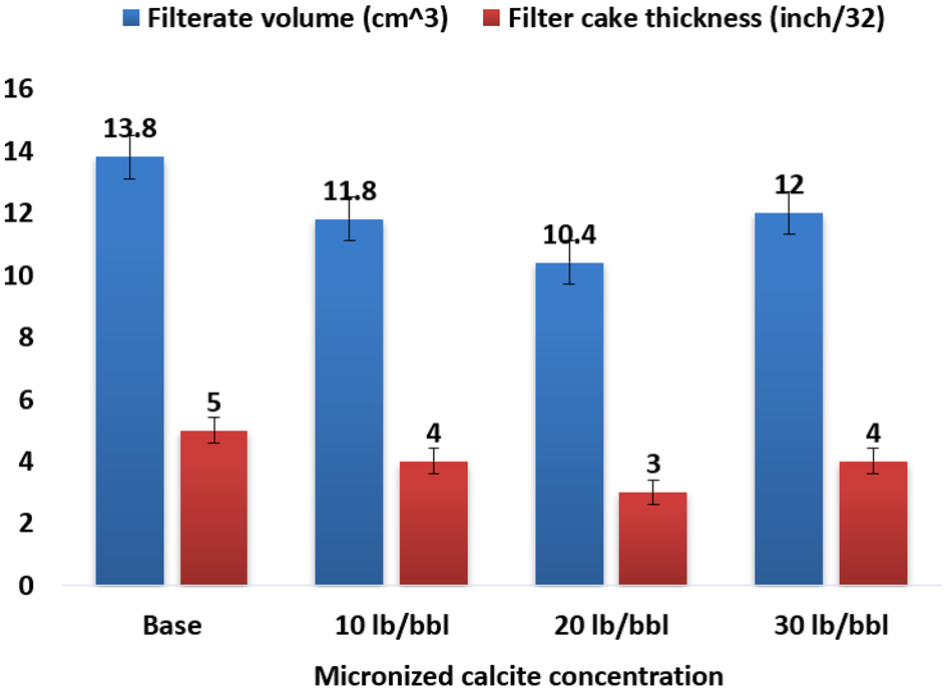
HPHT filtration results for different calcite microparticle concentrations.

**Figure 9:**
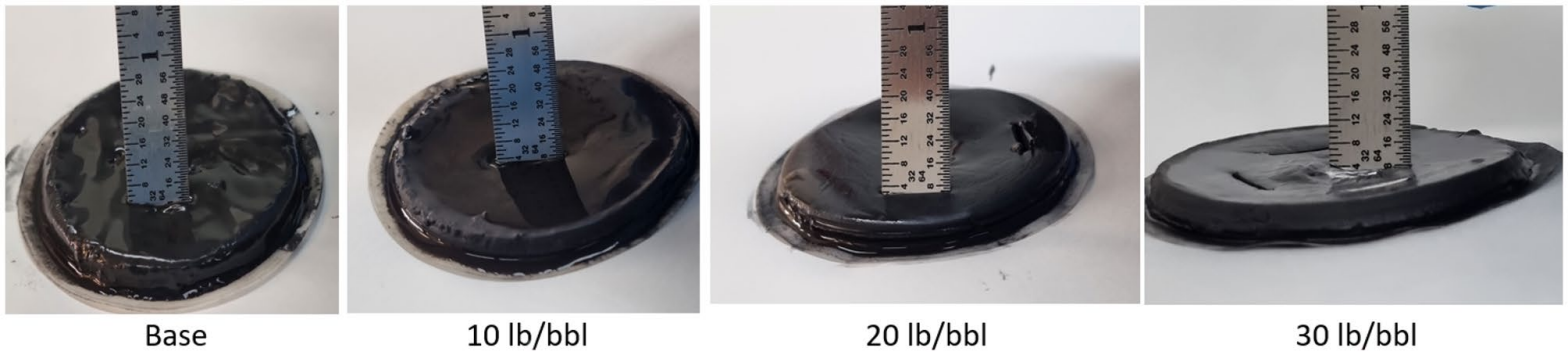
Filter cake thickness for different calcite microparticle concentrations.

The filtration properties of all samples were assessed and compared against the base fluid, and the results are depicted in Figs. 8 and 9. The introduction of increasing concentrations of micronized calcite demonstrated effective control over HPHT fluid loss, resulting in reductions of 14.5%, 24.6%, and 13% in the filtrate volume for 10 lb/bbl, 20 lb/bbl, and 30 lb/bbl, respectively. Concurrently, the filter cake thickness exhibited a reduction of 20%, 40%, and 20%, respectively.

In our pursuit of achieving a thin, impermeable filter cake to minimize formation damage, ensure wellbore stability, and reduce high values of the torque and drag, it is evident that the optimum micronized calcite concentration is 20 lb/bbl. However, the other two concentrations also enhance filtration properties as calcium carbonates act as a bridging agent, 20 lb/bbl is considered optimal in terms of both filtration volume and filter cake thickness.

**Figure 10:**
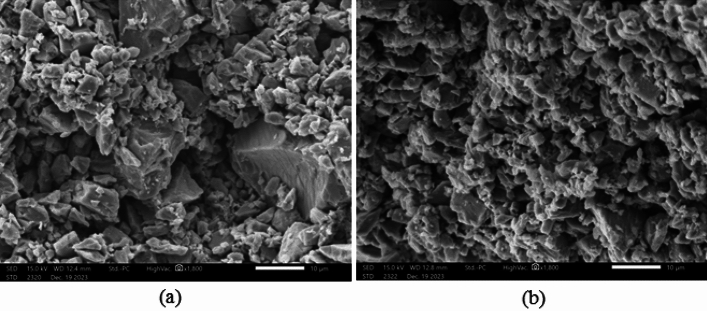
SEM of base fluid filter cake (**a**), and micronized calcite optimum concentration fluid filter cake (**b**).

To investigate the impact of micronized calcite on the structure of the filter cake, we analyzed both the filter cake formed with the base fluid and the filter cake with an optimized concentration, employing scanning electron microscopy (SEM). SEM enables the capture of high-resolution images, allowing for detailed observation of nanoscale features and providing insights into the surface topography of the filter cakes, as depicted in Fig. 10. Examination of the base fluid filter cake reveals substantial void spaces between the angular ilmenite particles as shown in Fig. 10a. In contrast, the filter cake incorporating micronized calcite demonstrates a distinct surface structure, wherein the micronized calcite particles effectively fill the gaps between the ilmenite particles. This results in a filter cake with reduced permeability and a smoother surface, as illustrated in Fig. 10b.

### Static sagging

**Figure 11:**
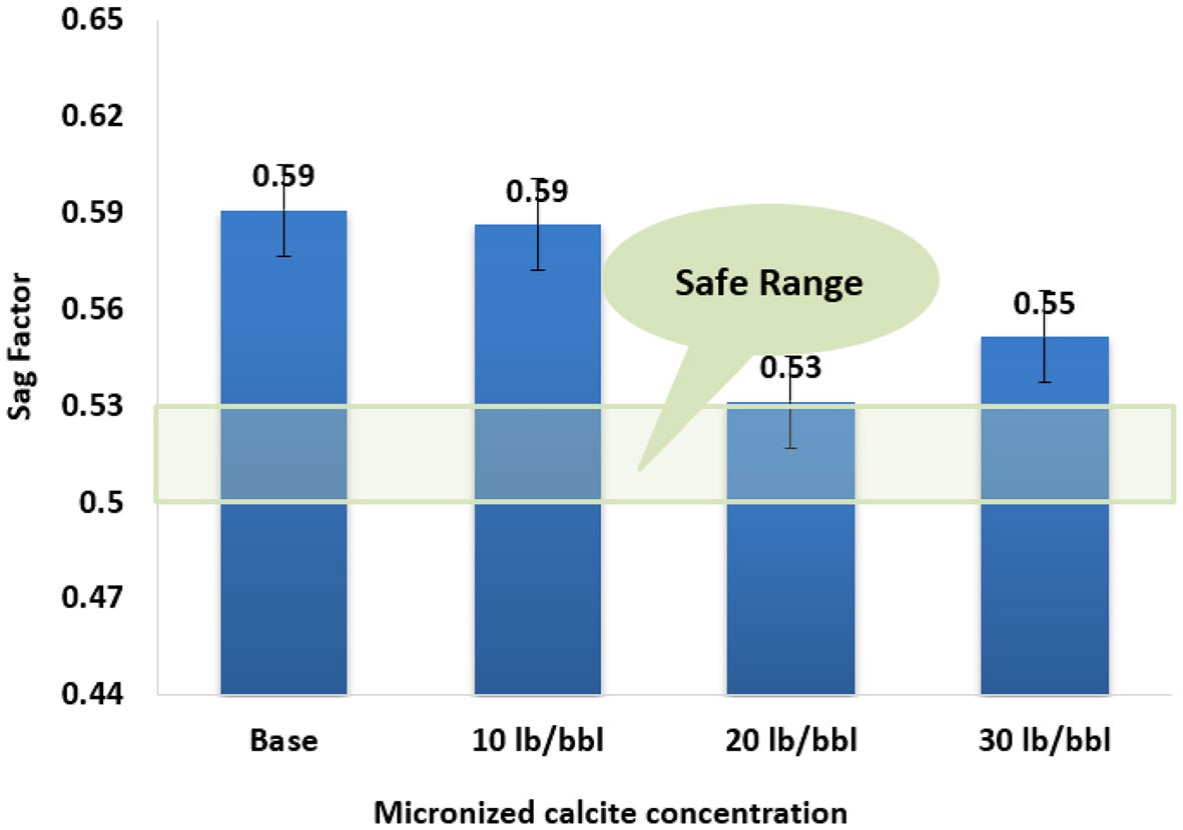
Static vertical sag test results for different calcite microparticles concentration.

The sag factor was calculated for each sample after 24 h of static conditions to assess the severity of ilmenite sagging, and the results are depicted in Fig. 11. The sag factor demonstrated a decrease of 0.6%, 10.3%, and 6.6% corresponding to concentrations of 10 lb/bbl, 20 lb/bbl, and 30 lb/bbl, respectively. Notably, the sample with 20 lb/bbl exhibited a sag factor of 0.53, indicating freedom from sagging issues. The attainment of a stable fluid devoid of sagging concerns directly impacts fluid rheology and filtrate properties, as previously discussed. It is crucial to avoid surpassing the optimum concentration of micronized calcite, as exceeding this level may introduce negative effects. At the optimum concentration of micronized calcite, due to the micronized calcium carbonate particles having a high surface area that acts to seal across the ilmenite particles, preventing them from settling down. Beyond this optimum concentration, the micronized calcite particles themselves begin settling due to their increased concentration.

## Conclusions

In this research, the impact of micronized calcium carbonates is analyzed as a potential additive for enhancing and optimizing ilmenite-weighted WBDF properties. Different concentrations of calcite microparticles, varied from 0, 10, 20, to 30 lb/bbl, were added to identical fluid formulations to study their effects. The main conclusions of that research can be highlighted as follows:

- The optimum concentration for incorporating calcite microparticles into the prepared ilmenite-weighted water-based drilling fluid is 20 lb/bbl, highlighting substantial improvements across all fluid properties.
- calcite microparticles prove to be a valuable additive for WBDF, demonstrating effectiveness under high-temperature and high-pressure conditions with no effect on the fluid pH.
- The inclusion of calcite microparticles positively impacts the rheological parameters of the fluid, with a substantial 37% improvement in yield point observed at the optimum concentration.
- calcite microparticles play a crucial role in controlling fluid loss, with reductions of 24.6% at the optimized concentration. Simultaneously, there is a decrease in filter cake thickness by 40%.
- At the optimum concentration of 20 lb/bbl of calcite microparticles, the sample ensures a stable condition concerning the sagging problem, whereas the other concentrations experience sagging issues.

## Data Availability

Individuals with a vested interest can seek permission to obtain the data directly from the corresponding author.

## Abbreviations

HPHT: High-pressure-high-temperature
AV: Apparent viscosity
PV: Plastic viscosity
YP: Yield point
WBDF: Water-based drilling fluid
OBDF: Oil-based drilling fluid
ppg: Pound per gallon
XRD: X-ray diffraction
XRF: X-ray fluorescence
SEM: Scanning electron microscopy
PSD: Particle size distribution
